# Photo Quiz

**DOI:** 10.3201/eid1703.101881

**Published:** 2011-03

**Authors:** Myron G. Schultz

**Affiliations:** Author affiliation: Centers for Disease Control and Prevention, Atlanta, Georgia, USA

**Keywords:** Tuberculosis, Robert Koch, discovery, tuberculosis and other mycobacteria, bacteriology, photo quiz

**Figure:**
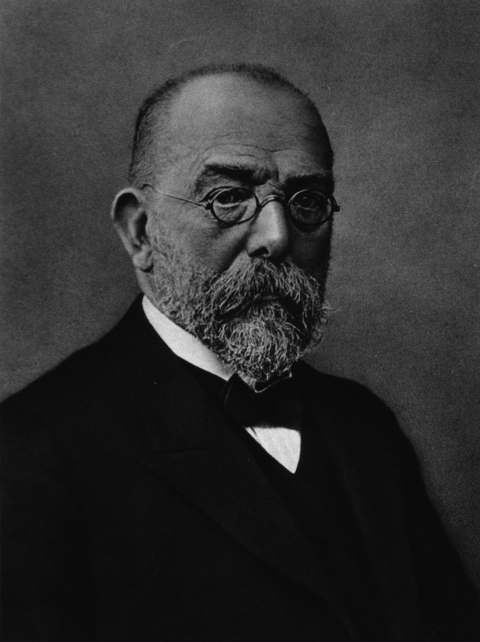
Who is this man?

Here is a clue: He discovered the organism that causes tuberculosis.A) Paul EhrlichB) Robert KochC) Louis PasteurD) Edward TrudeauF) Rudolf Virchow
