# Supplementary material for: Photo Quiz
Source: Emerg Infect Dis. 2011 Mar;17(3):547–9. doi: 10.3201/eid1703.101881 (PMC3298383; doi:10.3201/eid1703.101881)
Supplement: Related texts

# Other: Robert Koch

**DOI:** 10.3201/eid1703.101881

**Authors:** Myron G. Schultz

**Affiliations:** Author affiliation: Centers for Disease Control and Prevention, Atlanta, Georgia, USA

This is a photograph of Heinrich Hermann Robert Koch (1843–1910). Koch in Germany and Louis Pasteur in France were the 2 main founders of the science of bacteriology. Koch is best known for his discovery of *Mycobacterium tuberculosis*, the organism that causes tuberculosis. For this discovery, Koch was awarded the Nobel Prize in Physiology or Medicine in 1905.

Koch was born in Clausthal, Prussia, the son of a mining official. He was 1 of 13 children, 2 of whom died in infancy. At 5 years of age, he astounded his parents by telling them that he had, with the aid of newspapers, taught himself how to read. Koch studied medicine at the University of Göttingen under the German anatomist and pathologist Friedrich Gustav Jakob Henle (1809–1885); he graduated in 1866. Koch was influenced by Henle, who believed that infectious diseases were caused by living, parasitic organisms. For a brief period, Koch studied in Berlin, where he came under the influence of Rudolf Virchow. He then served in the Franco–Prussian War and later became a district medical officer in Wollstein, Prussian Poland.

Koch’s first major contribution, in 1876, was identification of the anthrax bacillus. Working as a provincial physician in the little town of Wollstein, far from other researchers, Koch used homemade equipment that he had invented to describe the entire life history of anthrax. He showed that the bacillus he found in blood samples could not survive for long outside a host but that it could exist for long periods under unfavorable circumstances in the form of spores. He originated a method of culturing the bacillus in the laboratory, outside the body of an infected animal.

In 1880, Koch’s work with anthrax earned him a position in the Imperial Health Office in Berlin. There he developed methods of isolating and cultivating disease-producing bacteria, and he formulated strategies for preventing their spread. In 1881, he published a report that advocated the need for pure culture for isolating disease-causing organisms and described in detail how to obtain them. His techniques for staining of bacilli, incubation, and pure culturing became the foundations of the science of bacteriology. He espoused the now-famous Koch’s postulates, conditions that are essential for establishing that an organism is the cause of a disease. Koch’s postulates state that an organism must be 1) found in all cases of the disease examined, 2) prepared and maintained in a pure culture, 3) capable of producing the original infection, even after several generations in culture, and 4) retrievable from an inoculated animal and cultured again.

In Berlin, Koch improved the methods that he had used while in Wollstein (e.g., staining and purification techniques) and bacterial growth media (e.g., agar plates and Petri dishes). With these techniques, Koch demonstrated the constant presence of the bacilli in the tuberculous lesions of men and animals; he cultivated these bacilli in pure culture on blood serum and produced tuberculosis at will by injecting the cultures into healthy animals. His findings were first presented in an article read before the Physiologic Society of Berlin on March 24, 1882. The logic, thoroughness, and importance of Koch’s presentation deeply impressed his audience, and this date has become a landmark in medical history; March 24 is now celebrated annually as World Tuberculosis Day. In 1883, Koch published a detailed description of his research, which was later translated into English under the title, The Etiology of Tuberculosis.

In 1883, while still busy working on tuberculosis, Koch was sent to Egypt as leader of the German Cholera Commission to investigate an outbreak of cholera. In Egypt, Koch discovered the vibrio organism that causes cholera and brought back pure cultures of it to Germany. He also studied cholera in India. Koch’s work on cholera, for which he received an award of 100,000 German marks**,** had strong influence because he advocated regular checks of water supplies and sewage disposal. Other diseases that Koch investigated in Italy, South Africa, India, and Indonesia included malaria, black water fever (etiologically related to malaria), plague, rinderpest, and trypanosomiasis.

Koch faltered from his usual perfectionism when he announced at the Tenth International Medical Congress in Berlin, in 1890, that he had found an inoculum that could protect against tuberculosis and even cure the established disease. It was referred to for a while as “Koch lymph” and later as “old tuberculin.” Koch’s testimony provoked tremendous enthusiasm that a cure for tuberculosis had been found. Unfortunately, the healing powers that he claimed for tuberculin were greatly exaggerated. Evidence accumulated that patients often became sicker after tuberculin injection. Opinion turned against the use of tuberculin and against Koch. In later years, tuberculin became useful as a diagnostic agent in the tuberculin skin test.

Koch also faltered on the question of whether tuberculosis in cattle was caused by the same organism as tuberculosis in man. He believed that bovine tuberculosis was not a danger to humans. He espoused his beliefs at several conferences in the United States and Britain. In 1901, at the London Congress on Tuberculosis, he encountered strong disagreement from leading bacteriologists, including Joseph Lister, Edmond Nocard, Bernhard Bang, John McFadyean, and Theobald Smith. Koch’s opinion had great influence because it was the testimony of an eminent authority. Yet, it was not backed by evidence. This modus operandi has led to many errors throughout medical history. In this instance, the consequences had enormous public health implications because this belief could have justified the use of tuberculous milk for human consumption. Within a few years, researchers, particularly Theobald Smith, and the English Royal Commission established beyond doubt that, contrary to Koch’s belief, bovine tuberculosis was a serious problem in humans, especially children. The campaigns for the pasteurization of milk and eradication of bovine tuberculosis were the practical outcomes of evidence-based research.

Throughout history, tuberculosis has been described under many names. In the 19th century its name evolved from phthisis to consumption and then, after Koch’s discovery, to tuberculosis. By whatever name, the amount of suffering brought about by tuberculosis is difficult to grasp. Wasting, fever, night sweats, breathlessness, pain in the side or shoulder, cough, abundance of sputum, and spitting of blood have long been associated with consumption. At the time of Koch’s discovery, consumption was a common cause of illness and death in Europe. Industrialization caused overcrowding of cities, and inhabitants suffered from malnutrition—the perfect scenario for the spread of tuberculosis. Koch believed that what he found in the laboratory should make a difference in the world. In the first article that he wrote on tuberculosis, he stated his lifelong goal, “I have undertaken my investigations in the interests of public health and I hope the greatest benefits will accrue therefrom.” Today, more than a century since Koch’s discovery, despite the many marvels of medicine and public health, tuberculosis is still one of the most common preventable disease of mankind, especially among the poor. Surely there is much more work to be done.

## References

[R-2-1] Brock TD. Robert Koch: a life in medicine and bacteriology. Washington: ASM Press; 1999.

[R-2-2] Dubos R. The white plague; tuberculosis, man, and society. 1st ed. New York: Little, Brown; 1952.

[R-2-3] Gradmann C. Laboratory disease: Robert Koch’s medical bacteriology. Baltimore: Johns Hopkins University Press; 2009.

[R-2-4] Koch R. Classics in infectious diseases. The etiology of tuberculosis: Robert Koch. Berlin, Germany 1882. Rev Infect Dis. 1982;4:1270–4. PubMeddoi:10.1093/clinids/4.6.12706818657

[R-2-5] Morens DM. At the deathbed of consumptive art. Emerg Infect Dis. 2002;8:1353–8 . PubMed10.3201/eid0811.02054912463180PMC2738548
